# Annotations

**Published:** 1901-04-27

**Authors:** 


### Annotations.

### Two Winter Pastimes.

With lengthening days we shall doubtless see the waning of a game which during the past winter has served to do something more than while away the idle hours. Ping-pong has been a god-send to many a stiffening joint and torpid liver, and to people approaching middle age the amount of exercise to be got out of so simple a game has been a revelation. The ping-pong player is at the mercy of his antagonist. However skilfully he may place his ball his opponent may send it into some strange corner to extricate it from which may involve many an unaccustomed genuflexion. And herein lies the virtue of the game. We are perhaps too prone to think that exercise is to be measured by effort. Effort may be laborious but yet confined to certain actions, whole groups of muscles being left idle—never contracted to the full, never stretched to the utmost—and there can be but little doubt that it is in the bending and the stooping and the unaccustomed posturing involved in the hunt for errant balls, quite as much as in the game itself, that the virtue of ping-pong lies as an indoor exercise for those approaching middle age. Another good winter pastime by which the young, especially, maintain suppleness of joint and elasticity of ligament, is properly ordered gymnastic exercise. It is more difficult, however, than some would think so to arrange gymnastics as to distribute the work in due proportion among the different muscles. As the virtue of ping-pong lies in the unexpected, so the vice of gymnastics, unless under proper supervision by qualified teachers, lies in its too orderly repetition. The importance of insisting on this is brought to mind by the remarks about skipping made by Lord Alverstone on the occasion of a recent gymnastic display at the Northampton Institute. He spoke in high praise of this form of exercise. But skipping is not altogether a good exercise for girls. By practising what may be described as "rising on the toes," so as to develop the calf and plantar muscles, much may be done to correct any tendency to flat foot, but by skipping the rebound is too great, and if carried on too long much injury may result. Skipping should only be undertaken as part of a system of exercises, never as an exercise by itself, and although it is good for little girls, there is a point in a young woman's growth when prolonged and competitive skipping may do serious harm. What we would insist on is that in all exercise monotonous repetition of the same movement is to be avoided. Of this all teachers of gymnastics are well aware. Unfortunately skipping is apt to be looked upon as quite a simple thing, not requiring supervision, and when girls try to "keep it up" then they hurt themselves.

### Isolation or Aggregation.

It is hardly to be wondered at that with the cost of hospital construction growing greater every year, and with the expense of hospital management in accordance with modern fashions and requirements increasing as it is doing, the question how far it is desirable to provide isolation hospitals for infectious diseases is becoming one of very serious importance to all sanitary authorities. We have already referred to the striking paper read by Dr. Killick Millard before the Society of Medical Officers of Health last February on the influence of hospital isolation in scarlet fever, and to the attacks on the whole system of isolation hospitals made from time to time by Dr. Marriott, of Nottingham, and we only mention them again because it is important that the whole subject should be kept constantly before our eyes, and that we should endeavour, by careful observation of the disease, each year to add some facts by which to explain the really puzzling condition in which the problem now stands. At first glance it seems so plain that every case of infectious disease which is isolated throughout its course means one less centre from which infection can become diffused, that, except for the expense involved in the process, sanitary authorities have had but little to urge against the plan of isolating as many cases as possible. But Dr. Millard's "appeal to statistics," however little it may clear up the question, does make this very obvious that it is by no means so simple a one as has been imagined. After a careful consideration of the many facts which he has collected, he concludes that the hospital isolation of scarlet fever (in towns) appears to have failed in materially reducing either the prevalence or the fatality of the disease, and that the fatality of the disease has fallen most in those large towns which have *not* practised hospital isolation! Here is indeed a nut to crack! It is clear that there is some factor engaged which has not yet been thoroughly worked out, something which in the "isolation towns" is working against the benefit which we can hardly help believing must result from isolation. What is this factor? Dr. Millard's explanation or hypothesis is that it is the aggregation of the cases (which has hitherto and of necessity accompanied their isolation in hospitals) which is at the bottom of the mischief, and he asks whether it is not conceivable that aggregation may be productive, in ways hitherto hardly suspected, of evils tending to neutralise the good which would otherwise have resulted; and that the type of the disease prevailing in towns where aggregation is practised, may have thus become modified in the direction of a higher fatality and a more persistent infectivity. We do not like this explanation. It is too vague, too intangible, and, we may add, too far from the facts. But for the trouble he has taken in collating the facts themselves, we think that Dr. Millard deserves the thanks of the profession, and possibly of the ratepayers. "Possibly" we say; for although some have thought that the facts will give the *coup de grace* to the system of isolation, it is by no means certain that they may not prove the excuse for a demand for a form of isolation still more expensive. If "isolation" has seemed to the ratepayer a scourging with snakes, "individual isolation" would indeed be a chastisement with scorpions.

### "Rubbish Tea."

A serious charge is being brought against the ingredients from which we brew the cup that cheers yet not inebriates, for we are told that the fine art of tea blending, by the proper exercise of which flavour and strength are judiciously combined, is being prostituted for the purpose of giving a false appearance of wholesomeness to rotten leaves. According to the methods of the modern planter, who makes large use of machinery, a good deal of tea is spoilt in the making, and it is easy to understand that the commercially successful blender is the one who succeeds in making his well-flavoured leaves bring him full value for as much of his "rubbish" also as may be. The seriousness of the matter is that the consumer, buying his pound or his half-pound at a time, is practically helpless in the matter. All he knows is that the tea does not agree with him. The stuff looks clean and well rolled, and in the packet or the handful it smells nice enough; but it is a mixture in which the rottenness of the rotten is covered up by the flavour and the aroma of the good. Mr. Brownen, F.C.S., in a letter to the *Times*, says that quite recently there has been a meeting of planters in Ceylon, at which it was stated that teas known to be unfit for human consumption had been exported, and he adds from his own knowledge that recent samples of tea obtained in this country contain leaves which, although dried, are perfectly rotten and full of the microbes of decomposition. He maintains that although the consumer cannot easily detect these defects in the blended article as he buys it, nothing could be simpler than for the Government to stop the importation of such stuff.

